# Investigating the association between neoplasms and MOG antibody-associated disease

**DOI:** 10.3389/fneur.2023.1193211

**Published:** 2023-06-09

**Authors:** Milena Trentinaglia, Alessandro Dinoto, Sara Carta, Vanessa Chiodega, Sergio Ferrari, Vincenzo Andreone, Giorgia Teresa Maniscalco, Sara Mariotto

**Affiliations:** ^1^Neurology Unit, Department of Neurosciences, Biomedicine, and Movement Sciences, University of Verona, Verona, Italy; ^2^Neurological Clinic and Stroke Unit, Naples, Italy; ^3^Multiple Sclerosis Center, Naples, Italy

**Keywords:** myelin oligodendrocyte glycoprotein antibody-associated disease (MOGAD), paraneoplastic neurological syndrome, tumor, cancer, immune checkpoint inhibitors

## Abstract

**Introduction:**

The association of myelin oligodendrocyte glycoprotein (MOG) antibody associated disease (MOGAD) and tumors has seldom been reported. We aim to investigate the occurrence of tumors in a cohort of patients with MOGAD and to describe their clinical features, in addition to previously reported cases.

**Methods:**

We retrospectively identified patients with MOGAD (i.e., compatible clinical phenotype and positive MOG antibodies analysed with a live cell-based assay) from 1/1/2015 to 1/1/2023 who had a neoplasm diagnosed within 2  years from MOGAD onset. Furthermore, we performed systematic review of literature to identify previously reported cases. Clinical, paraclinical and oncological findings were collected and reported as median (range) or number (percentage).

**Results:**

Two of 150 MOGAD patients (1%) had a concomitant neoplasm in our cohort. Fifteen additional cases were retrieved from literature. Median age was 39 (16–73) years-old, 12 patients were female. ADEM (*n* = 4;23.5%), encephalomyelitis (*n* = 3;17.6%), and monolateral optic neuritis (*n* = 2;11.8%) were the most frequent phenotypes. Median number of treatments was 1 (range 1–4), improvement was reported in 14/17 cases (82.4%). Oncological accompaniments were teratoma (*n* = 4), CNS (*n* = 3), melanoma (*n* = 2), lung (*n* = 2), hematological (*n* = 2), ovary (*n* = 1), breast (*n* = 1), gastrointestinal (*n* = 1), and thymic (*n* = 1) neoplasms. Median time from tumor diagnosis to MOGAD onset was 0 (range − 60 to 20) months. MOG expression in neoplastic tissue was reported in 2/4 patients. Median PNS-CARE score was 3 (range 0–7): 11 patients were classified as “non-PNS,” 5 as “possible PNS,” and 1 as “probable PNS.”

**Discussion:**

Our study confirms that MOG is a low-risk antibody for paraneoplastic neurological syndromes and that the clinical presentation and oncological accompaniments are extremely variable. Most of these patients were classified as non-PNS, whereas only a minority was diagnosed with possible/probable PNS, frequently in association with ovarian teratoma. These findings support the notion that MOGAD is not a paraneoplastic disease.

## Introduction

Paraneoplastic neurological syndromes (PNS) are defined by the presence of specific clinical features in association with cancer and specific autoantibodies (1). Among these autoantibodies, those targeting aquaporin-4 (AQP4) and myelin oligodendrocyte glycoprotein (MOG) are associated with a low risk of cancer, even though MOG or AQP4 expression may be detected on cancer tissue, supporting a paraneoplastic origin in those few reported cases. While the association between neoplasms and AQP4-seropositive neuromyelitis optica spectrum disorder (NMOSD) has been investigated in previous studies (2–4), data on patients affected by MOG antibody-associated disease (MOGAD) (5) are still lacking and limited on few case reports (6). Aim of this study is to report the association between neoplasms and MOGAD in a single-center cohort and, additionally, to provide a systematic evaluation of previously reported cases.

## Materials and methods

We retrospectively identified patients with MOGAD [i.e with a compatible phenotype and positive MOG-Abs analysed with a live cell-based assay as previously described (7)] and a neoplasm diagnosed within 2  years from disease onset. Clinical, paraclinical, and oncological data were collected and are herein reported. As cancer screening is currently not required in patients with MOGAD, paraneoplastic analyses were performed by treating physicians on an individualized basis according to physical examination or routine laboratory screening. In addition, to identify previously reported cases of paraneoplastic MOGAD, a systematic literature review based on PubMed/Medline database was performed (March 7th, 2023) using the following research queries: (“myelin oligodendrocyte glycoprotein” OR “MOG”OR “MOG-EM” OR “MOGAD”) AND (“cancer” OR “paraneoplastic” OR “tumor” OR “teratoma”) including studies on individual cases. Furthermore, all articles included after abstract screening were cross-referenced. Relevant reported clinical, paraclinical, and oncological findings were collected in an electronic database. PNS Care score was evaluated in each case at clinical presentation, as previously described (1).

A descriptive statistical analysis was performed using median (range) and number (%), as appropriate (IBM SPSS 26) including both data obtained from the retrospective study and the systematic review. For estimating the percentage of cancer occurring within 2 years in patients with MOGAD, we included only cases identified in our cohort excluding those obtained from the systematic review. Finally, patients classified as non-PNS were compared to patients fulfilling the criteria of Probable/Possible/Definite PNS (Fisher’s and U-Mann Whitney tests). Patients gave their informed consent for being included in this study.

## Results

### Retrospective cohort study

Of 185 patients diagnosed with MOGAD from 1/1/2015 to 1/1/2023, two patients out of 150 with available clinical information had a neoplasm within 2  years from MOGAD onset (~1%). These two cases are reported below. Both reported cases resulted negative for additional autoantibodies analysed with commercial and on in-house tissue-based assay (8).

#### Case #1

A 59-year-old man presented with acute onset of fever, imbalance, dysarthria, and hallucinations. Brain MRI was unremarkable, while on CSF analysis pleocytosis (645 cells/mm^3^ N.V. <5) and increased protein levels (477 mg/dL N.V. <45 mg/dL) were observed. Although an extensive infectious and autoimmune screening including antibodies to neuronal and cell surface antigens were negative, the clinical suspicion of brainstem encephalitis led to start treatment with steroids, with complete recovery. Respectively two and 9  months after onset, generalized tonic–clonic seizures occurred and antiepileptic drugs (AED) were commenced. A repeated brain MRI showed multiple T2/FLAIR hyperintense lesions in the supratentorial and infratentorial white matter, with pons contrast enhancement. 4  months later, the patient developed a severe paraparesis with sensory level and sphincter dysfunction. Total body computed tomography (CT) scan was unremarkable while spinal cord MRI revealed multiple T2/FLAIR hyperintensities (at C7-D1, D8-D10, and D11 level) suggestive of mixed short and longitudinally extensive transverse myelitis (LETM). A repeated CSF analysis showed slightly increased cell count (7/mm^3^), normal protein values (41.8 mg/dL), and negative oligoclonal bands (mirror pattern). An expanded autoimmune screening with live cell-based assay revealed the presence of serum MOG-Abs (titre 1:320). The patient was diagnosed with MOGAD and treated with high dose intravenous steroids followed by slow tapering. Since only a partial response was observed, treatment with Rituximab was commenced. 20  months later, the appearance of a unilateral axillary lymphadenopathy led to a diagnosis of a non-Hogdkin lymphoma and the patient was treated with chemotherapy.

#### Case #2

**Figure 1:**
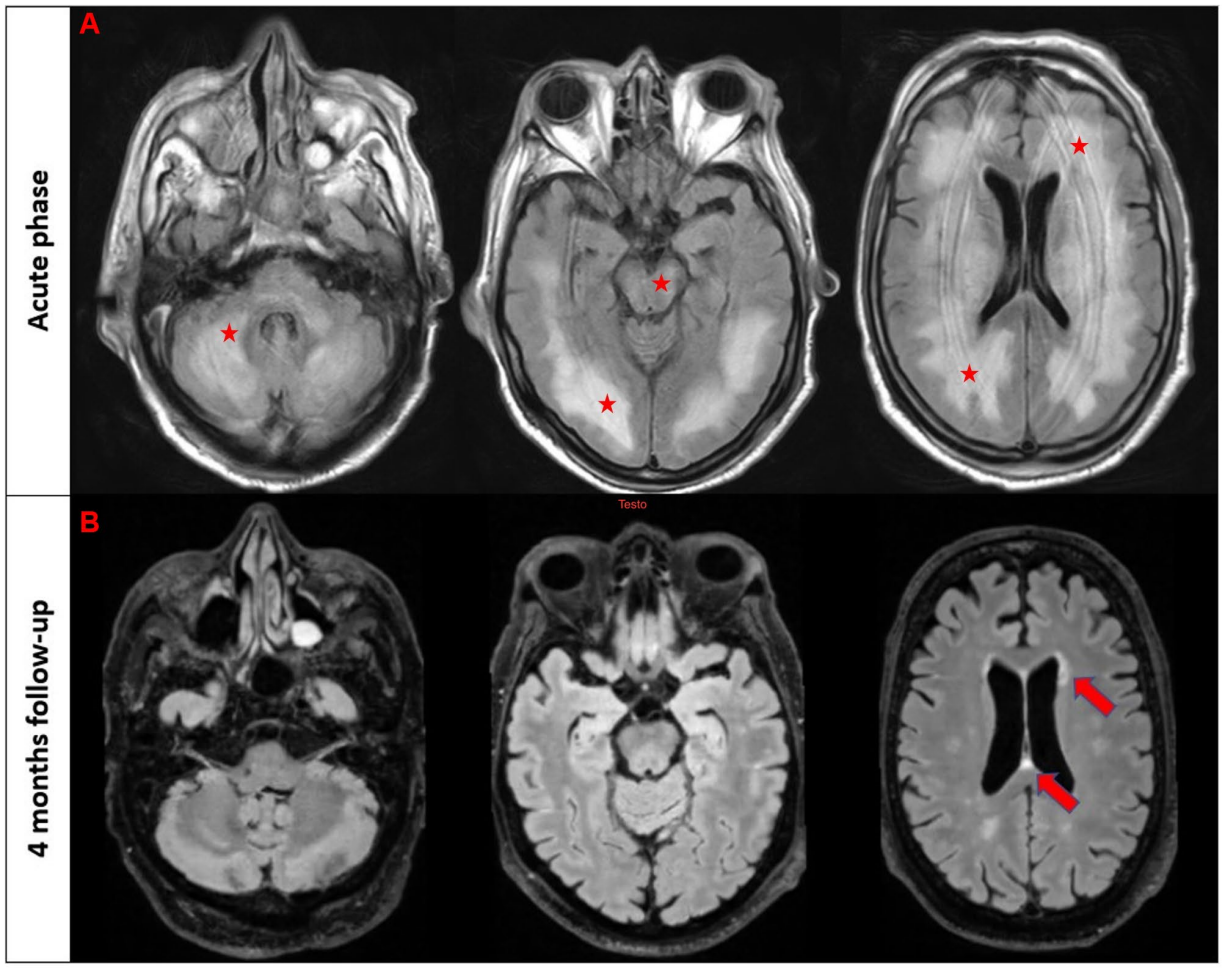
T2/FLAIR imaging in the acute phase (first raw) and follow-up brain MRI after 4  months in patient #2. The first three images from the acute phase reveal a diffuse T2/FLAIR hyperintensity involving bilateral white matter, brainstem, and cerebellum (marked with stars). The follow-up scans demonstrate a nearly complete resolution of these abnormalities with few scattered white matter hyperintensities in the periventricular regions (marked with an arrow).

A 64-year-old man while admitted for pneumonia and acute kidney failure developed subacute consciousness impairment requiring orotracheal intubation. On neurological examination he was comatose and unresponsive to pain stimulus with right-sided pyramidal signs. Brain MRI showed diffuse T2/FLAIR hyperintense lesions in the supratentorial and infratentorial white matter involving the brainstem (Figure 1A). EEG revealed generalized theta and delta slowing without epileptic abnormalities. CSF analysis demonstrated mild pleocytosis (10 cells/mm^3^). An extensive screening for CNS infections, autoimmune/paraneoplastic encephalitis, metabolic encephalopathy, and uremic hemolytic syndrome yielded negative results except for the detection of serum MOG-Abs (titer 1:320) using a live cell-based assay. The patient was diagnosed with MOGAD-related encephalitis and treatment with high dose intravenous steroids followed by oral tapering led to significant neurological improvement. Clinical inspection revealed a skin lesion localized in the right hemithorax suggestive for malignancy. Thus, a biopsy was performed and histological analysis confirmed a locally invasive melanoma (stage pT1b). Total body CT as well as sentinel lymph node biopsy excluded systemic localization. 4  month later the patient was asymptomatic with a normal neurological evalution, MOG-Abs titers were decreased (1:160), and a control MRI showed almost complete resolution of pre-existent lesions (Figure 1B).

### Systematic review

Of 616 results, 10 studies underwent to full-text evaluation and 8 were included in the final analysis. Furthermore, 7 studies were included through cross-referencing of relevant articles. A total number of 15 studies with 15 cases were included in the final synthesis [(9–23). The PRISMA flow chart is reported in the Supplementary materials.

### Clinical features of the pooled cohort

In the pooled cohort analysis 17 cases were included (Table 1). Median age at onset was 39 years (range 16–73) and 12 patients were female (70.5%). Clinical phenotypes were consistent with: ADEM (*n* = 4, 23.5%), encephalomyelitis (*n* = 2, 11.8%), monolateral optic neuritis (*n* = 2, 11.8%) and one (5.9%) of each of bilateral optic neuritis, encephalitis, encephalomyeloradiculitis, isolated myelitis, myelitis and bilateral optic neuritis, myelitis and bilateral optic neuritis with brainstem involvement, optic neuritis and brainstem syndrome, optic neuritis and meningoencephalitis, brainstem syndrome, encephalitis and myelitis. One patient was also positive for anti-GFAP and anti ITPR−1 antibodies and presented with combined CNS and PNS involvement, which has been previously reported in MOGAD (24, 25). Patients received a median of 1 acute treatment (range 1–4): steroids were administered in 16 cases, intravenous immunoglobulins in 3, plasma exchange in 3, and rituximab in 3. Clinical improvement after immunotherapy was reported in 14 patients (82.4%), whereas no improvement was observed in 3 cases (17.6%). Regarding the oncological accompaniments, associated neoplasms were teratoma (*n* = 4, in one case with also ganglioneuroma), CNS tumors (*n* = 3, pituitary macroadenoma, meningioma, and astrocytoma), melanoma (*n* = 2), lung cancer (*n* = 2, both adenocarcinoma), hematological malignancies (*n* = 2, non-Hodgkin lymphoma and cutaneous T cell lymphoma), borderline tumor of the ovary (*n* = 1), ductal breast carcinoma (*n* = 1), colon adenocarcinoma (*n* = 1), and thymic hyperplasia (*n* = 1). MOG protein expression in the excised tumoral tissue was reported in 2/4 patients (50%). Median time from tumor diagnosis to MOGAD onset was 0  months (range from −60 to +20, with negative values indicating that tumor preceded the onset of the neurological syndrome). Oncological treatment included surgery in 11 cases, chemotherapy in 4, radiotherapy in 1, other treatments in 3 (including hormonal therapy, pembrolizumab, and anti EGFR-TKI). Three patients did not receive any treatment. Median PNS-CARE score was 3 (range 0–7). In particular, 11 patients were classified as “non-PNS,” 5 patients as “possible PNS,” and 1 patient as “probable PNS.” The comparison between patients with non-PNS and with possible and probable PNS did not yield any significant difference, with the exception for a trend favoring the association with ovarian teratoma (*p* = 0.063), Table 2.

**Table 1:** Demographic, clinical, and oncological features of the included cohort.

Study	Sex/age	Neurological manifestation	Tumor association	MOGAD onset in relation to tumor	Neurological treatments	Tumor treatment	Neurological outcome	MOG expression on tumor	PNS-CARE score/ final diagnosis	Other antibodies
Herein presented case (nr. 1)	M/59	Brainstem syndrome, followed by supratentorial encephalitis, myelitis	Non-Hodgkin lymphoma	20 months prior	Steroids, RTX	CHT	Improvement	N.A.	(2 + 0 + 1) 3/non-PNS	No
Herein presented case (nr. 2)	M/64	Encephalitis	Melanoma	Concomitant	Steroids	Surgery	Improvement	N.A.	3 (2 + 0 + 1)/non-PNS	No
Li et al.	F/49	Myelitis, bilateral optic neuritis, and brainstem syndrome	Lung adenocarcinoma	1 month after	Steroids	Surgery, anti EGFR-TKI	Improvement	N.A.	1 (0 + 0 + 1)/non-PNS	No
Cherian et al.	F/37	Myelitis	Ductal breast carcinoma	12 months after	Steroids	Surgery, CHT, Hormones	Improvement	N.A.	3 (2 + 0 + 1)/non-PNS	No
Rodenbeck et al.	F/64	ADEM	Lung carcinoma	Concomitant	Steroids, PLEX	N.A.	No improvement	N.A.	(0 + 0 + 1)/non-PNS	No
Jarius et al.	F/18	ADEM	Teratoma+Ganglioneuroma	2 months after	Steroids, PLEX, IvIg	Surgery	Improvement	N.A.	4 (0 + 0 + 4)/possible-PNS	No
Kwon et al.	M/38	Myelitis and bilateral optic neuritis	Cutaneous T-cell lymphoma	6 months prior	Steroids	CHT	Improvement	Negative	1 (0 + 0 + 1)/non-PNS	No
Cohen et al.	F/52	ADEM	Colon adenocarcinoma with lung metastases	5 years after adenocarcinoma	Steroids	N.A.	No improvement	N.A.	0 (0 + 0 + 0)/non-PNS	No
Wildemann et al.	F/26	Optic neuritis	Teratoma	11 months after	Steroids	Surgery	Improvement	Positive	4 (0 + 0 + 4) /possible-PNS	No
Cirkel et al.	F/44	Encephalomyeloradiculitis	Borderline tumor of the ovary	Concomitant	Steroids, IvIg, RTX, PLEX	Surgery	No improvement	Negative	(3 + 0 + 1)4/possible-PNS	GFAP, ITPR-1
Ajam et al.	M/25	Optic neuritis	Meningioma	3 months after	Steroids	Surgery	Improvement	N.A.	1 (0 + 0 + 1)/non-PNS	No
Delgado et al.	F/37	Bilateral optic neuritis	Pituitary macroadenoma	Concomitant	Steroids	None	Improvement	N.A.	1 (0 + 0 + 1)/non PNS	No
Zhang et al.	F/61	Encephalomyelitis	Teratoma	Concomitant	Steroids	Surgery	Improvement	Positive	7 (3 + 0 + 4)/probable-PNS	No
Cobo-Calvo et al.	F/16	ADEM	Teratoma	Concomitant	IvIg	Surgery	Improvement	N.A.	4 (0 + 0 + 4)possible-PNS	No
Liu et al.	M/39	Encephalomyelitis	Melanoma	6 months after	Steroids	Surgery+pembrolizumab	Improvement	N.A.	4 (3 + 0 + 1)/possible-PNS	No
Zhong et al.	F/49	Optic neuritis and brainstem syndrome	Astrocytoma	4 years after	Steroids	Surgery+RT	Improvement	N.A.	0 (0 + 0 + 0)/non-PNS	No
Hurtubise et al.	F/18	Optic neuritis and meningoencephalitis	Thymic hyperplasia	3 months prior	Steroids, RTX	None	Improvement	N.A.	1 (0 + 0 + 1)/non-PNS	No

M, male; F, female; ADEM, acute disseminated encephalomyelitis; RTX, rituximab; PLEX, plasma exchange; IvIg, intravenous immunoglobulins; PNS, paraneoplastic neurological syndrome; CHT, chemotherapy; RT, radiotherapy; GFAP, glial fibrillart acid protein; ITPR-1, inositol trisphosphate receptor-1.

**Table 2:** Comparison of patients according to the PNS-CARE classification.

	Non-PNS (*n* = 11)	Possible/probable PNS (*n* = 6)	value of *p*
Age (years)	49 (18–64)	32.5 (16–61)	0.256
Sex (female)	7 (63.6%)	5 (83.5%)	0.365
Clinical phenotype	ADEM 2 (18.2%) Bilateral optic neuritis 1 (9.1%) Encephalomyelitis 1 (9.1%) Myelitis 1 (9.1%) Myelitis and bilateral optic neuritis 1 (9.1%) Myelitis, bilateral optic neuritis, and brainstem syndrome 1 (9.1%) Monolateral optic neuritis 1 (9.1%) Optic neuritis + meningoencephalitis 1 (9.1%) Optic neuritis and brainstem syndrome 1 (9.1%)	ADEM 2 (33.3%) Encephalomyelitis 2 (33.3%) Encephalomyeloradiculitis 1 (16.7%) Monolateral optic neuritis 1 (16.7%)	0.676
Associated tumor	CNS 3 (27.3%) Hematological 2 (18.2%) Lung 2 (18.2%) Gastrointestinal 1 (9.1%) Breast 1 (9.1%) Melanoma 1 (9.1%) Thymus 1 (9.1%)	Teratoma 4 (66.7%) Ovary 1 (16.7%) Melanoma (16.7%)	*0.063*
Time from MOGAD to tumor diagnosis	0 (−60–20)	-1 (−11–0)	0.733
Number of treatments	1 (1–2)	1 (1–4)	0.660
Neurological outcome (improvement)	9 (81.8%)	5 (83.3%)	0.728

ADEM, acute disseminated encephalomyelitis; PNS, paraneoplastic neurological syndrome; CNS, central nervous system; MOGAD, myelin oligodendrocyte antibody-associated disorder.

## Discussion

**Figure 2:**
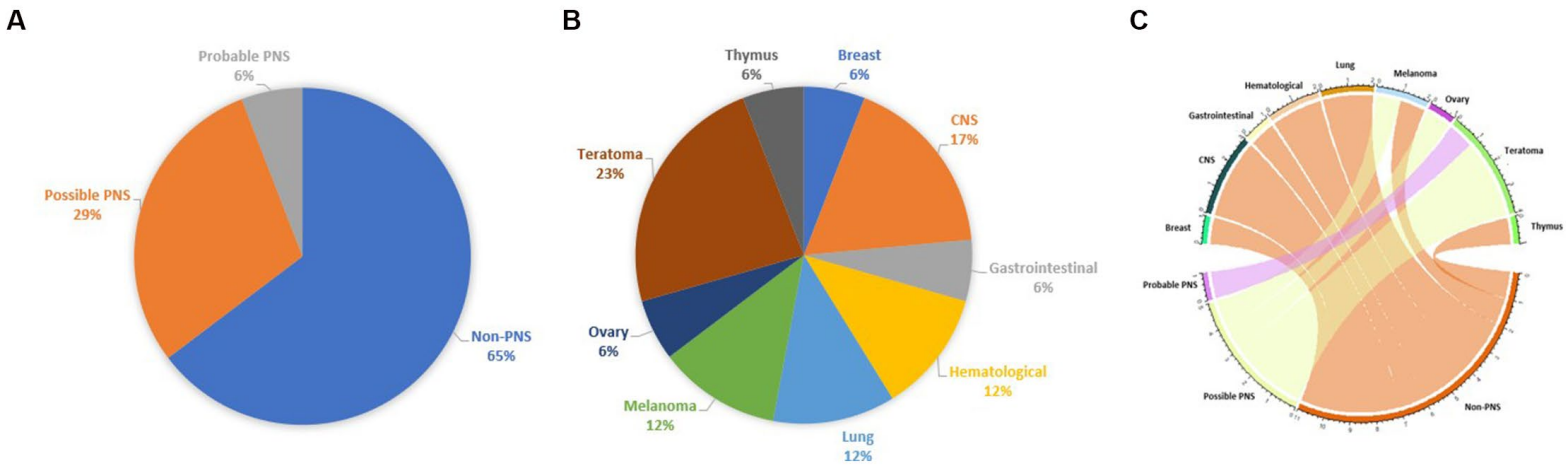
Chart representing the percentage of each tumor in the pooled cohort analysis **(A)**, the fulfillment of PNS diagnostic criteria **(B)**, and a circos plot represents the association between neoplasms and fulfillment of PNS diagnostic criteria **(C)**.

Our study supports the weak association between MOGAD and tumors, since: (a) the prevalence of neoplasms in a cohort of patients with MOGAD is low, (b) oncological accompaniments are extremely variable and MOG is usually not expressed in neoplastic tissue, (c) most of reported cases do not fulfill the criteria of PNS; (d) there are no striking features that could distinguish cases with possible/probable PNS from those with non-PNS, with the notable exception for a trend favoring the presence of ovarian teratoma in the first group (Figure 2).

In comparison with a previous study (14) reporting 11.3% of patients with a history of cancer within 12  months from MOGAD onset, with higher rates of prevalence in elderly patients, we observed a significant lower number (1%) of MOGAD cases associated with tumors. In addition, we found a significant lower number of patients with concomitant neoplasms and MOGAD (6.5% vs. 35%). These discrepancies may be explained by the differences in terms of population groups and inclusion criteria.

In support of the absence of a strict association between MOGAD and tumors by systematically reviewing the published literature we observed that neoplasms associated with MOGAD are extremely variable and include even some non-malignant tumors, which mostly fell in the non-PNS group. Even though we did not find an association between MOGAD and a specific cancer, the presence of ovarian teratoma seems to be particularly relevant, as both tumors that expressed MOG protein were teratomas (17, 18), and these data may support a paraneoplastic origin. Furthermore, the presence of teratomas has been associated with different antibody-mediated CNS disorders including AQP4 positive NMOSD (26) and anti-NMDAR encephalitis (27). Consistently, even though no patients were diagnosed with definite PNS, those who had a diagnosis of probable/possible PNS had a statistical trend favoring the presence of an underlying teratoma when compared to non-PNS patients. Consistently, PNS-CARE score was mostly driven by the presence of an underlying teratoma which, alone, would give a score of 4, leading to a diagnosis of possible PNS regardless of the clinical phenotype. This finding could suggest that PNS-CARE scoring system may present some limitations in the setting of a condition characterized by heterogeneous clinical presentations and very specific oncological accompaniments. Of note, one patient without an ovarian teratoma who fulfilled the criteria of possible PNS was receiving treatment with the immune checkpoint inhibitor pembrolizumab, which probably triggered MOGAD. Accordingly, iatrogenic demyelination as an immune-related adverse event of cancer immunotherapy is rare but should not be overlooked.

Finally, we did not find any specific clinical phenotype or demographic feature which may suggest a paraneoplastic trigger and thus should prompt an oncological screening. Furthermore, most of patients improved with immunotherapy only, regardless of neoplasm removal, which is atypical for PNS (28). We previously suggested to perform a paraneoplastic screening regardless of clinical presentation in AQP4 positive NMOSD despite the lack of specific clinical features in suspected paraneoplastic NMOSD, since several cases of paraneoplastic NMOSD have been reported and AQP4 tumor expression can occur even in patients with atypical oncological accompaniments (as non-adenocarcinomas) (2). On the contrary, we do not support tumor screening in MOGAD, since the lack of cancer expression beyond ovarian teratoma and the lack of other suggestive features do not support this extensive screening.

Our study presents several limitations including (a) the small sample size, the absence of a control group, and the unavailability of a systematic and uniform screening of neoplasms in our cohort, (b) the lack of evaluation of MOG expression in many studies, including our cases, and (c) the potential reporting bias favoring the overreporting of patients presenting with MOGAD and neoplasms.

Despite these limitations, our study confirms that MOG is a low-risk antibody for paraneoplastic neurological syndromes and that the clinical presentation and oncological accompaniments are extremely variable. These findings support the notion that MOGAD is not a paraneoplastic disease.

## Data availability statement

The raw data supporting the conclusions of this article will be made available by the authors, without undue reservation.

## Ethics statement

The studies involving human participants were reviewed and approved by the Ethics Committee of Verona University Hospital. The patients/participants provided their written informed consent to participate in this study.

## Author contributions

MT and AD equally contributed and collected data, and wrote the first draft of the manuscript. GM and SM shared the senior co-authorship. All the authors performed a revision to the manuscript, gave the critical intellectual content, contributed to the article, and approved the submitted version.

## Conflict of interest

The authors declare that the research was conducted in the absence of any commercial or financial relationships that could be construed as a potential conflict of interest.

## Publisher’s note

All claims expressed in this article are solely those of the authors and do not necessarily represent those of their affiliated organizations, or those of the publisher, the editors and the reviewers. Any product that may be evaluated in this article, or claim that may be made by its manufacturer, is not guaranteed or endorsed by the publisher.
