# Supplementary material for: Investigating the association between neoplasms and MOG antibody-associated disease
Source: Front Neurol. 2023 Jun 9;14:1193211. doi: 10.3389/fneur.2023.1193211 (PMC10289293; doi:10.3389/fneur.2023.1193211)
Supplement: Supplementary file 1 [file Data_Sheet_1.DOCX]

Supplementary Figure 1 - PRISMA flow chart of the study.

**Identification of studies via other methods**

**Identification of studies via databases and registers**

Records identified from:

Cross-reference (n = 7)

Records identified from:

Databases (n = 616)

**Identification**

Records screened

(n = 616)

Records excluded

(n = 605)

Reports sought for retrieval

(n = 7)

Reports sought for retrieval

(n = 11)

**Screening**

Reports assessed for eligibility

(n = 7)

Reports excluded:

No individual patient data (n = 1)

Reports assessed for eligibility

(n = 11)

Reports excluded:

Not relevant (n=2)

Studies included in review

(n = 15)

**Included**

*From:*  Page MJ, McKenzie JE, Bossuyt PM, Boutron I, Hoffmann TC, Mulrow CD, et al. The PRISMA 2020 statement: an updated guideline for reporting systematic reviews. BMJ 2021;372:n71. doi: 10.1136/bmj.n71. For more information, visit: <http://www.prisma-statement.org/>
